# Physiological Measurements and Transcriptome Survey Reveal How Semi-mangrove *Clerodendrum inerme* Tolerates Saline Adversity

**DOI:** 10.3389/fpls.2022.882884

**Published:** 2022-07-15

**Authors:** Minting Liang, Feng Hu, Dongsheng Xie, Zhibin Chen, Qingzhi Zheng, Qiyun Xie, Feng Zheng, Dongming Liu, Shuguang Jian, Hongfeng Chen, Xuncheng Liu, Faguo Wang

**Affiliations:** ^1^Key Laboratory of South China Agricultural Plant Molecular Analysis and Genetic Improvement and Guangdong Provincial Key Laboratory of Applied Botany, South China Botanical Garden, Chinese Academy of Sciences, Guangzhou, China; ^2^Department of Landscape and Tourism Planning and Design, Guangzhou Urban Planning and Design Survey Research Institute, Guangzhou, China

**Keywords:** salt tolerance, halophyte, *Clerodendrum inerme*, physiological response, transcriptome reprogramming

## Abstract

Salinity adversity has been a major environmental stressor for plant growth and reproduction worldwide. Semi-mangrove *Clerodendrum inerme*, a naturally salt-tolerant plant, can be studied as a successful example to understand the biological mechanism of saline resistance. Since it is a sophisticated and all-round scale process for plants to react to stress, our greenhouse study interpreted the response of *C. inerme* to salt challenge in the following aspects: morphology, osmotic protectants, ROS production and scavenging, ion homeostasis, photosynthetic efficiency, and transcriptome reprogramming. The results drew an overview picture to illustrate the tolerant performance of *C. inerme* from salt acclimatization (till medium NaCl level, 0.3 mol/L) to salinity stress (high NaCl level, 0.5 mol/L). The overall evaluation leads to a conclusion that the main survival strategy of *C. inerme* is globally reshaping metabolic and ion profiles to adapt to saline adversity. These findings uncover the defense mechanism by which *C. inerme* moderates its development rate to resist the short- and long-term salt adversity, along with rebalancing the energy allocation between growth and stress tolerance.

## Introduction

Among various abiotic stresses, salinity is one of the major environmental factors affecting the geographical distribution of plants and reducing the productivity of crops (Zhu, 2016). Approximately 7% of the world's land is affected by either salinity or sodium toxicity, and the production of over 30% of irrigated crops is limited by salinity stress (Schroeder et al., 2013). Halophytes, also known as salinity tolerant plants, representing only 2% of terrestrial plant species, have evolved diverse strategies to survive in salinity (Glenn et al., 1999). Understanding the adaptive strategies of halophytes to salt stress will be extremely important for us to cultivate salt-tolerant plants.

The salt sodium chloride (NaCl) is the main cause of salt stress in plants. The high concentration of Na^+^ causes consecutively adverse impacts on plants. First, the accumulation of Na^+^ limits water uptake and nutrient absorption, which induces primary stresses including osmotic stress and ionic stress. Second, the primary stresses lead to oxidative stress, which is defined as excess production of reactive oxygen species (ROS). Over-accumulated ROS causes a series of secondary stresses in plant cells such as reducing cell division, inhibiting photosynthesis, and damaging cellular components, which eventually impedes plant growth (Zhu, 2002, 2016).

As sessile organisms, plants have evolved diverse physiological strategies to defend saline environments and adjust growth under high salt conditions. Plants can scavenge excessive ROS for detoxification by the antioxidant systems including superoxide dismutase (SOD), peroxidase (POD), ascorbate peroxidase (APX), catalase (CAT), glutathione peroxidase (GPX), and peroxiredoxin (PrxR). Plants also advantageously use ROS as an important messenger in response to high salinity (Takahashi and Asada, 1988; Mittler et al., 2004; Dietz et al., 2006; Zhu, 2016). Maintenance of the intracellular K^+^ and Na^+^ homeostasis is important for the activities of many cytosolic enzymes. Plants restore ion homeostasis by removing Na^+^ from the cytoplasm *via* Na^+^/H^+^ antiporters, which confers better salinity tolerance (Zhu, 2003; James et al., 2006; Park et al., 2016; Bose et al., 2017). Moreover, abundant osmolytes like proline, glycine-betaine, trehalose, and sugar alcohols were produced by plants in salinity. These compounds adjust the osmotic pressure of the cytosolic compartment to alleviate salt-induced water deficiency (Flowers et al., 2015; Munns and Gilliham, 2015).

In recent years, extensive investigations of salt stress responses using transcriptomic approaches have been characterized for various halophytes. It was demonstrated that transcripts related to membrane transport, osmoprotection, redox metabolism, or protein synthesis expressed differentially in *Beta vulgaris* subsp. *maritima* to saline adversity (Skorupa et al., 2016). In *Glehnia littoralis*, genes encoding transcription factors or involved in multiple signaling pathways, such as plant hormone, calcium, and phospholipase, were responsive to salinity conditions (Li et al., 2018). Besides, expression pattern cluster analysis revealed that genes related to secondary metabolic pathway and transcription were enriched significantly in sweet sorghum RIO during salt imposition (Chen et al., 2022). Moreover, in Guar *Cyamopsis tetragonoloba* (L.) Taub., genes associated with stress-signaling pathways, transporters, chromatin remodeling, microRNA biogenesis, and translational machinery play primary roles to tolerate salt stress (Acharya et al., 2022).

*Clerodendrum inerme*, a halophyte in the family Lamiaceae, usually grows on coastal beaches as an important semi-mangrove plant. In Pakistan, it has been scheduled as a hydro-halophyte used in saline agriculture while in China it was listed as a medicinal halophyte for many diseases (http://www.grhc.sdnu.edu.cn/) (Khan and Qaiser, 2006). Although the salt tolerance mechanisms of *C. inerme* are unclear, it is an ideal salt-tolerant material to study its great agricultural and scientific values. In this study, we quantified and interpreted the physiological and biochemical responses of *C. inerme* to different salt concentrations, such as growth inhibition, developmental changes, metabolic adaptations, photosynthetic acclimation, and ionic homeostasis. We also investigated the rapid and long-term transcriptomic changes of *C. inerme* to salt exposure. Our findings evaluate an overall defense and tolerance strategy of *C. inerme* overcoming salt adversity, which will be helpful to guide salinity-tolerant plant engineering in the future.

## Results

### Plant Growth in Response to Saline Stress

**Figure 1:**
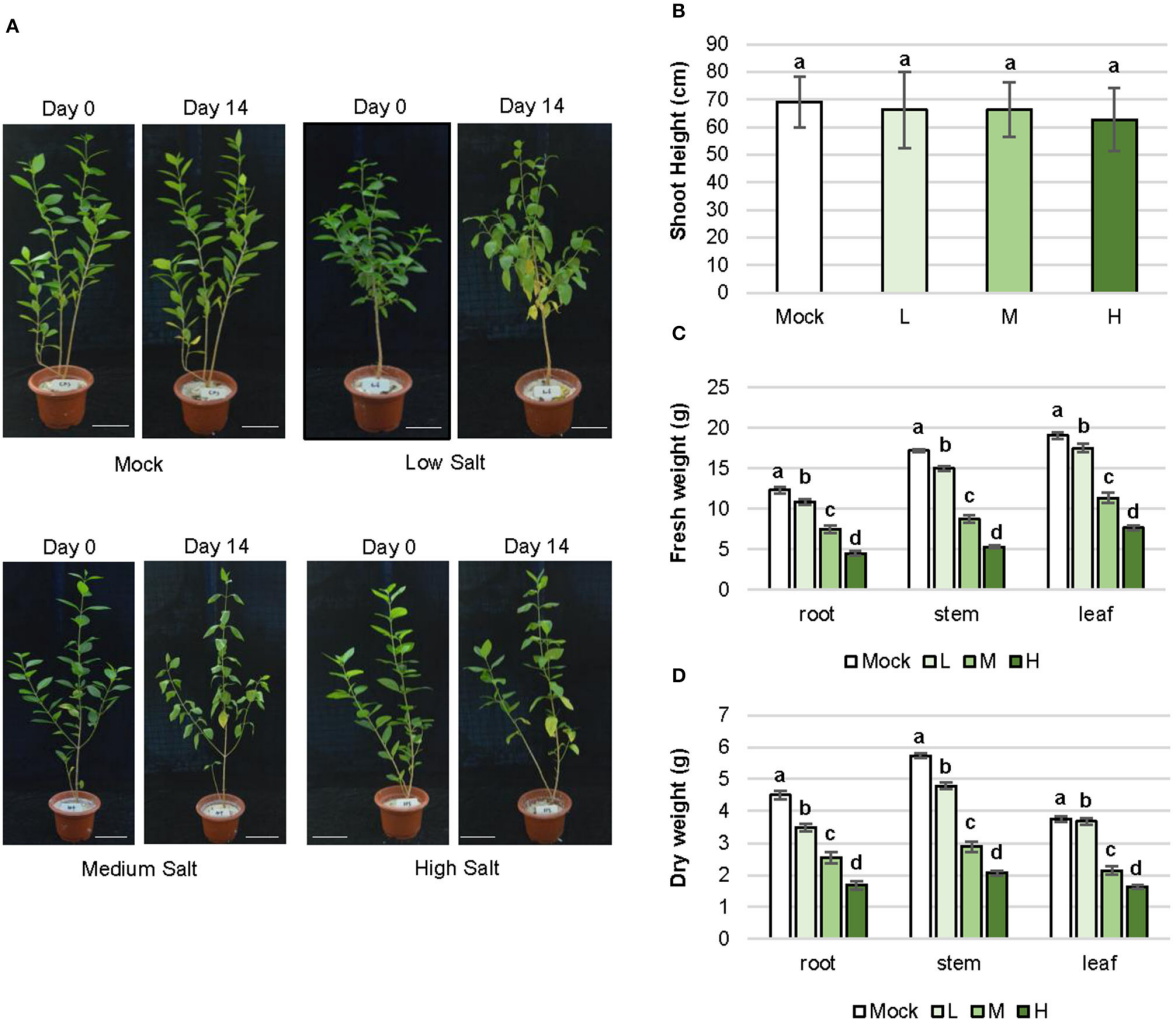
Morphogenesis and biomass alteration of *C. inerme* in response to salinity stress. **(A)** Plant architecture of *C. inerme* on day 0 and day 14 after corresponding salt treatment. Shoot height **(B)**, fresh weight **(C)** and dry weight **(D)** of root, stem and leaf respectively on day 14 under NaCl stress. Different letters indicate significant differences among plant groups (*p* < 0.05, Mann Whitneytest). Mock, no salt control; L, low salt stress; M, medium salt stress; H, high salt stress.

The growth state of the *C. inerme* plant visibly reflects tolerance to salt stress. Low (0.1 M), medium (0.3 M), and high (0.5 M) concentrations of NaCl were set for saline stress treatment, according to the saline concentration of seawater in plant habitat and the maximum salt content range that *C. inerme* can bear (Lotfi et al., 2010; Rad et al., 2021). Inhibition of plant height was not observed on day 14 in the presence of NaCl at different experimental concentrations, yet more yellow leaves appeared and premature defoliation was observed with the increase in salt level (Figures 1A,B; Supplementary Figure 1A). Although *C. inerme* exhibited more yellow sagged leaves at low NaCl concentrations compared to mock control, the yellow leaves barely fell off. However, leaves turned yellow, softened, deformed, and shed rapidly under medium and high salt concentrations, leaving only fewer leaves at the base of the plant. The growth response to saline stress was also detected *via* biomass accumulation. The fresh and dry weights of root, stem, and leaf decreased gradually with the aggravation of NaCl adversity (Figures 1C,D). Under high salt stress, the fresh and dry weights of root were 63.73 and 62.68% lower than the control, respectively. Accordingly, fresh and dry weights of stem were 68.33% and 63.95% lower than those of control, respectively. A similar reduction was also observed in leaf. The remarkable decrease in biomass was likely due to arrested development. Consistent with the general stress response, under medium and high concentration salt adversity, *C. inerme* distributes more biomass to roots to utilize more metabolites and energy to resist the stress environment around the root with an increased root-to-shoot ratio by dry weight (Supplementary Figure 1B).

### Alleviation of Intracellular Oxidative Stress Caused by Salinity

**Figure 2:**
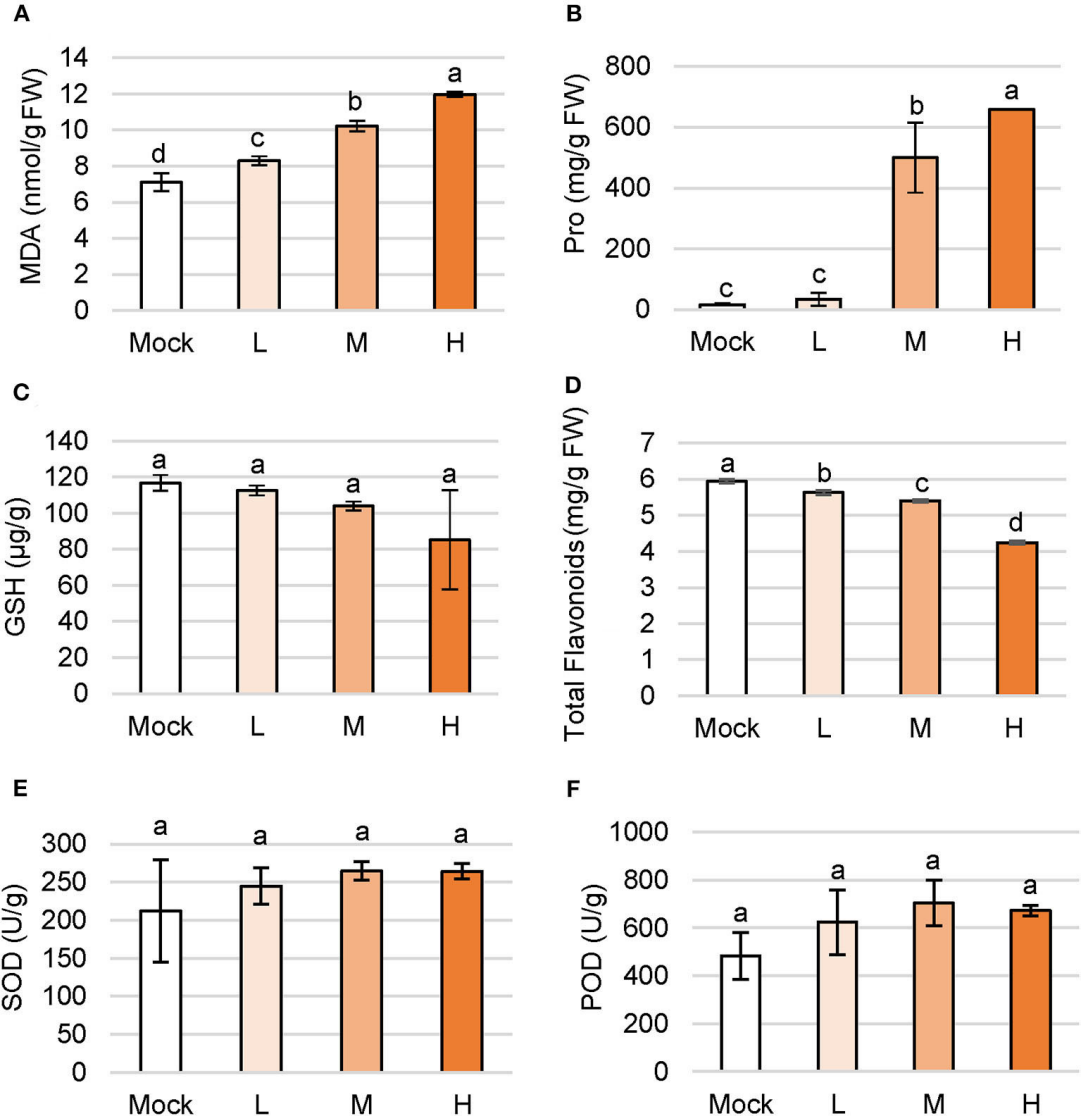
Changes of primary and secondary metabolites in *C. inerme* under salinity stress. Content of MDA **(A)**, proline **(B)**, GSH **(C)**, total flavonoids **(D)**, SOD **(E)** and POD **(F)** in plant leaves on day 14 under salt adversity. Different letters indicate significant differences among plant groups (*p* < 0.05, Mann Whitney test). Mock, no salt control; L, low salt stress; M, medium salt stress; H, high salt stress.

Plants rapidly accumulate ROS to activate defense response to environmental stress and MDA is also produced to aggravate the lesion of the membrane system, which is an important index to detect the degree of damage in plant cells (Meloni et al., 2003). The MDA level gradually increased in accordance with the concentration of NaCl treatment. Under low, medium, and high salt treatment, the MDA content in plants were 16.6, 43.7, and 68.3% higher than the control group, respectively (Figure 2A).

Proline (Pro) is ubiquitous in plants and acts as a regulator to maintain the osmotic homeostasis across the cell membrane. Content of Pro increased with the aggravation of salt stress, and a higher level of Pro showed stronger osmotic regulation. Accumulation of Pro content in *C. inerme* leaves boosted with the concentration of the saline solution. The Pro levels were 500.6 mg/g and 658.6 mg/g under medium and high salt treatments, that is, 2,895.2 and 3,840.4% higher than the mock control (Figure 2B). To sum up, osmotic stress occurred under low salt stress, but Pro began to increase dramatically under medium salt stress, suggesting the strong Pro regulation ability of *C. inerme*.

Glutathione (GSH) is a kind of γ-tripeptide consisting of glutamate, cysteine, and glycine with amide bonds and thiol groups, namely the most widely distributed non-protein sulfhydryl antioxidant in cells. The GSH content of *C. inerme* was maintained from 85.31 to 116.71 μg/g, showing no significant difference under various levels of saline stress, indicating the increase of ROS in the *C. inerme* plant caused by NaCl unlikely affecting the biosynthesis of GSH (Figure 2C).

The low molecular secondary metabolite flavonoids not only regulate the growth of plants but also reflect strong antioxidant properties. The accumulation of flavonoids improves stress resistance and reduces oxidative damage. Content of total flavonoids in *C. inerme* decreased gradually with the aggravation of saline stress. Under high salt stress, total flavonoids were left to 4.24 mg/g, which decreased by 28.57% compared with that in the control treatment (Figure 2D). The result suggests that the flavonoids of *C. inerme* were greatly affected by the salt imposition.

SOD is the first defense line of ROS scavenging in the antioxidant enzyme system, which disproportionates O^2−^ to H_2_O_2_. SOD scavenges ROS and peroxides together with POD and CAT. SOD activity of *C. inerme* maintained between 212.45 and 264.58 U/g among groups (Figure 2E), indicating that these concentrations of saline solution influenced little on SOD activity in this halophyte. Correlatively, no significant effect on POD activity was observed under indicated salt treatment (Figure 2F), which together suggests that the strength of saline adversity was below the sensitivity of the antioxidant enzyme system in the long-term salinity aggression, or was already adapted by this salt-tolerant plant.

### Photosynthetic Performance to the Salt Stress

**Figure 3:**
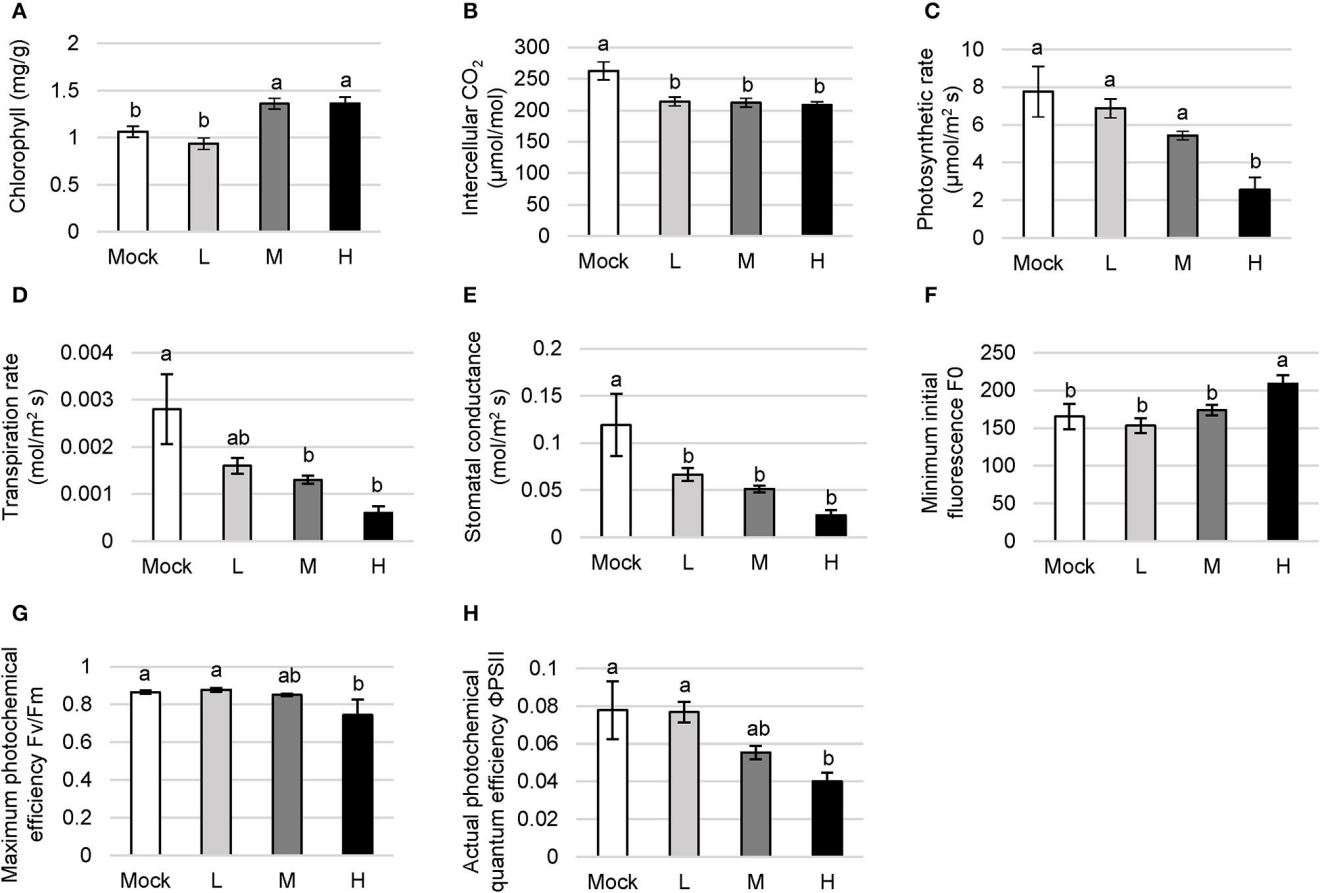
Photosynthetic function in *C. inerme* to the saline adversity. Content of chlorophyll **(A)**, photosynthetic rate **(B)**, transpiration rate **(C)**, intercellular CO_2_ level **(D)**, stomatal conductance **(E)**, F0 **(F)**, Fv/Fm **(G)** and ΦPSII **(H)** of *C. inerme* leaves were determined on day 14 to different levels of NaCl stress. Different letters indicate significant differences among plant groups (*p* < 0.05, Mann Whitney test). Mock, no salt control; L, low salt stress; M, medium salt stress; H, high salt stress.

Chlorophyll is the key pigment for photosynthesis, directly determining the photosynthetic rate and the ability of assimilation in plants. Salt-sensitive plants decompose chlorophyll under salt stress whereas salt-tolerant ones continue to maintain the biosynthesis of chlorophyll or even tend to enhance this process. *C. inerme* showed a decreasing chlorophyll level under low salt adversity with a reduction of 11.89% compared with no salt control (Figure 3A). However, the chlorophyll content climbed and saturated to 1.36 mg/g when treated with the medium and high saline conditions, increasing by 28.02% compared with no salt treatment, in agreement with its halophyte properties.

The intercellular CO_2_ content indicates CO_2_ storage in plant cells. It directly affects the stock of substrates for photosynthesis, and yet the absorption against the environmental CO_2_ through guard cells. The CO_2_ content gradually declined with the increase in salt concentration (Figure 3B). The net photosynthetic rate directly reflects the CO_2_ assimilation capacity of the unit leaf area in the plant. Low and medium salt adversity lead to a subtle decrease, whereas high salt treatment caused a significant reduction in the photosynthetic rate of *C. inerme* compared with the mock control. The photosynthetic rate was down to 2.56 mol/m^2^s under high NaCl, reduced by 67.04% compared with mock treatment (Figure 3C).

The usage and transportation of water by the plant can be indicated *via* transpiration rate. The transpiration rate in *C. inerme* decreased with the increase of salt concentration, and dropped to 0.0006 mol/m^2^s under high salinity, counting for 78.57% lower than control. The highest transpiration rate was 0.0028 mol/m^2^s without NaCl but showed no significant difference compared with that under low salinity conditions (Figure 3D). The degree of stomata opening was reflected by stomatal conductance, and directly affects the photosynthesis, respiration, and transpiration in the plant. Correlatively, the stomatal conductance of *C. inerme* peaked to 0.1190 mol/m^2^ s under no salinity while dropped to 0.0233 mol/m^2^ s when treated with the highest level of NaCl, which was 80.42% lower than no salt control (Figure 3E).

Photosystem II (PSII) is an important component of the thylakoid membrane and its destruction under adversity directly affects the photosynthetic rate. F0 represents the intensity of chlorophyll fluorescence emission and reflects the heat dissipation protection mechanism of PSII. The *C. inerme* showed similar F0 under no, low, or medium salt conditions, except in the presence of high salinity F0 peaked to 208.93, which was 26.48% higher than that of no NaCl control (Figure 3F). The result suggests that PSII was lightly destroyed under high salinity stress and its potential activity was inhibited.

Fv/Fm reflects the maximum photochemical efficiency of PSII to use light energy and electron transfer. Under low and medium salinity, Fv/Fm showed no difference compared to control (Figure 3G). Fv/Fm under high NaCl treatments was 0.74, which was 13.98% lower than the mock control. Besides, ΦPSII represents the actual photochemical quantum efficiency of PSII in plants under the light. It shows the ratio of excitation energy used for photochemical pathways to the total excitation energy entering PSII, which is an important indicator for plant photosynthetic capacity. ΦPSII in *C. inerme* decreased gradually with the increase of salt concentration (Figure 3H). Like Fv/Fm, there was no significantly different ΦPSII among no, low, or medium salinity stress. However, the ΦPSII value decreased remarkably under high salt treatment, which was 48.59% lower than control, suggesting the inhibition of photosynthetic rate as well as certain damage to the photosynthetic apparatus. Collectively, these data reveal that the photosynthetic system of *C. inerme* was damaged to some extent by the salinity, especially under high salinity stress.

### Dynamic Change of Cations Flow Under NaCl Adversity

**Figure 4:**
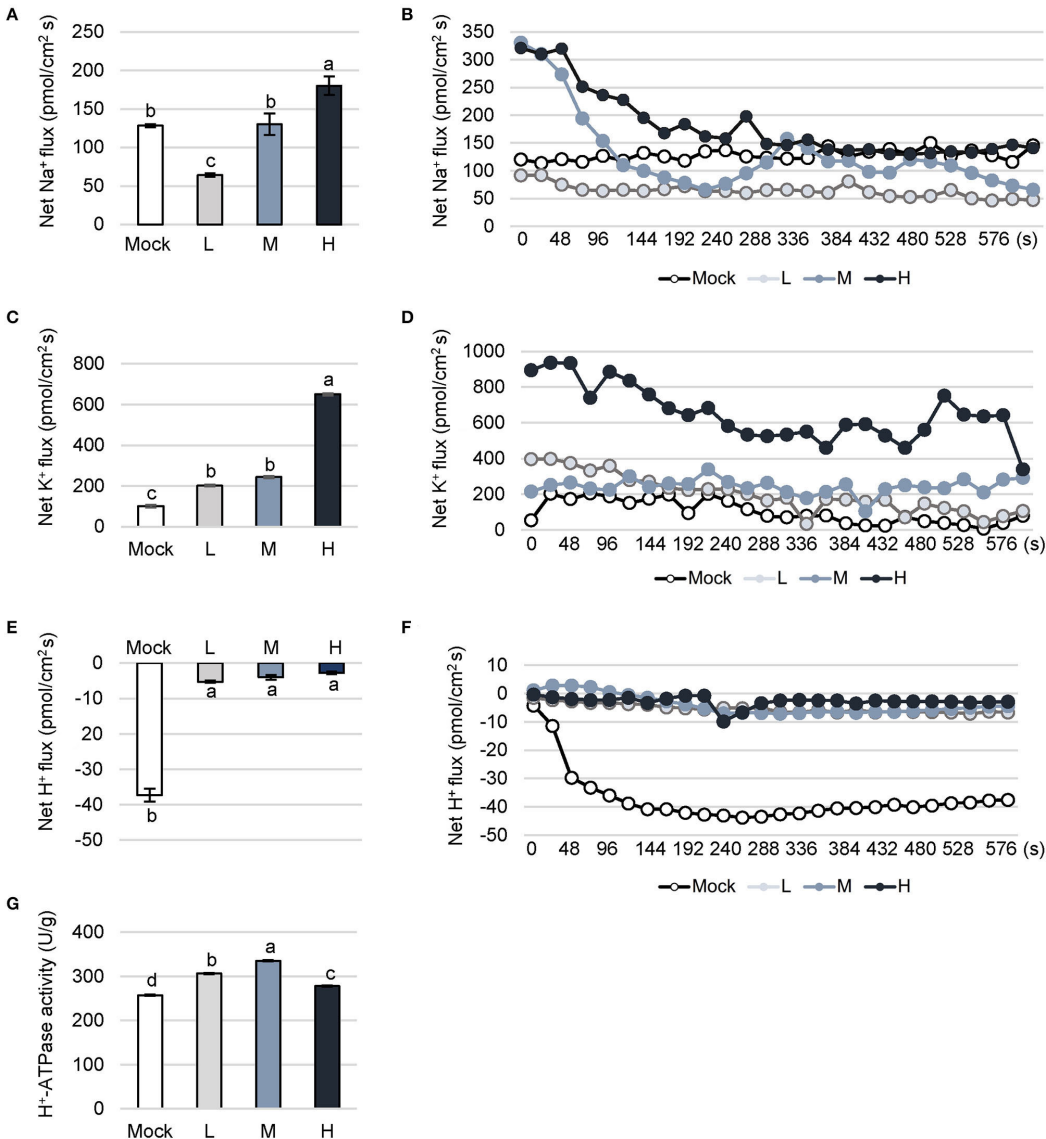
Dynamic fluctuation of cation flow under different levels of salt stress. Na^+^ **(A,B)**, K^+^ **(C,D)** and H^+^ **(E,F)** flux and activity of H^+^-ATPase **(G)** in *C. inerme* leaves were detected on day 14 under different saline stress. Different letters indicate significant differences among plant groups (*p* < 0.05, Mann Whitney test). Mock, no salt control; L, low salt stress; M, medium salt stress; H, high salt stress.

Plant cells accumulate Na^+^ in response to salt stress. Excessive Na^+^ triggers peroxidation on plasma membranes, and seriously affects cellular transport. Terrestrial plants have evolved a complex mechanism to reduce the entry of Na^+^ by the selective absorption of K^+^. Redundant Na^+^ is then pumped out of the cytoplasm through the Na^+^/H^+^ antiporter on the tonoplast and isolated in the vacuole. In this study, the measurement is equally divided into 26 time points with 10 min. Beyond this time range, the value is unreliable due to serious plant tissue damage. The column chart showed the average of the values at 26 time points. The ions outflow when the value is above zero and ions flow into cells when the value is below zero. Na^+^ flux was significantly decreased in *C. inerme* plants under a low level of saline compared to mock plants (Figures 4A,B). However, no difference was detected between medium saline treatment and control. Whereas, Na^+^ flux increased to 180.18 pmol/cm^2^s under high NaCl, counting for 1.4 times of mock control.

K^+^ plays a vital role in the osmotic adjustment of plant resistance to stress. K^+^ competes with Na^+^ to bind some sites on the plasma membrane. Moreover, Na^+^ accumulation stimulates K^+^ influx to the cell, and in turn, the influx of K^+^ promotes the efflux of Na^+^ under salt stress. In *C. inerme*, K^+^ flux climbed to 201.78 pmol/cm^2^s and 243.37 pmol/cm^2^s in the presence of low or medium salt stress, which were significantly higher than no salt control (Figures 4C,D). Under high salinity stress, K^+^ flux increased to 650.67 pmol/cm^2^s, which was 6.4 times that of control. It is speculated that the ion channels of the plasma membrane in the presence of high salt levels are significantly activated.

Dynamic change of H^+^ is related to the efflux of Na^+^ for plant stress resistance. The H^+^-ATPase on the plasma membrane and tonoplast provides energy for the reverse transport of Na^+^/H^+^. The H^+^ influx of *C. inerme* under control increased gradually and finally saturated (Figures 4E,F). Under different levels of salinity, H^+^ showed an influx much lower than control, basically maintaining between 2.73 and 5.24 pmol/cm^2^s. Besides, H^+^-ATPase locates on the plasma membrane and plays an important role in ion homeostasis. H^+^-ATPase provides the energy for the Na^+^/H^+^ antiporter on the membrane, pumping H^+^ out of the cytoplasm to form an H^+^ electrochemical potential gradient to facilitate the efflux of Na^+^. Increased H^+^-ATPase activity is a sign to reflect the plant salt tolerance and decreased one shows irreversible damage to the plasma membrane. The results showed the decrease in H^+^ influx and the increase in outflow reflects the enhancement of Na^+^/H^+^ antiporter and H^+^-ATPase. The activity of H^+^-ATPase in *C. inerme* under low and medium salt stress were 306.5 and 335.74 U/g, which were 19.15and 30.51% higher than control, separately (Figure 4G). Under high NaCl stress, the activity of H^+^-ATPase dropped to the level between low and no salt treatment, suggesting slightly decreased activity of this enzyme in response to high adversity.

### Transcriptional Reprofiling in the Short- and Long-Term Response to Salt

**Figure 5:**
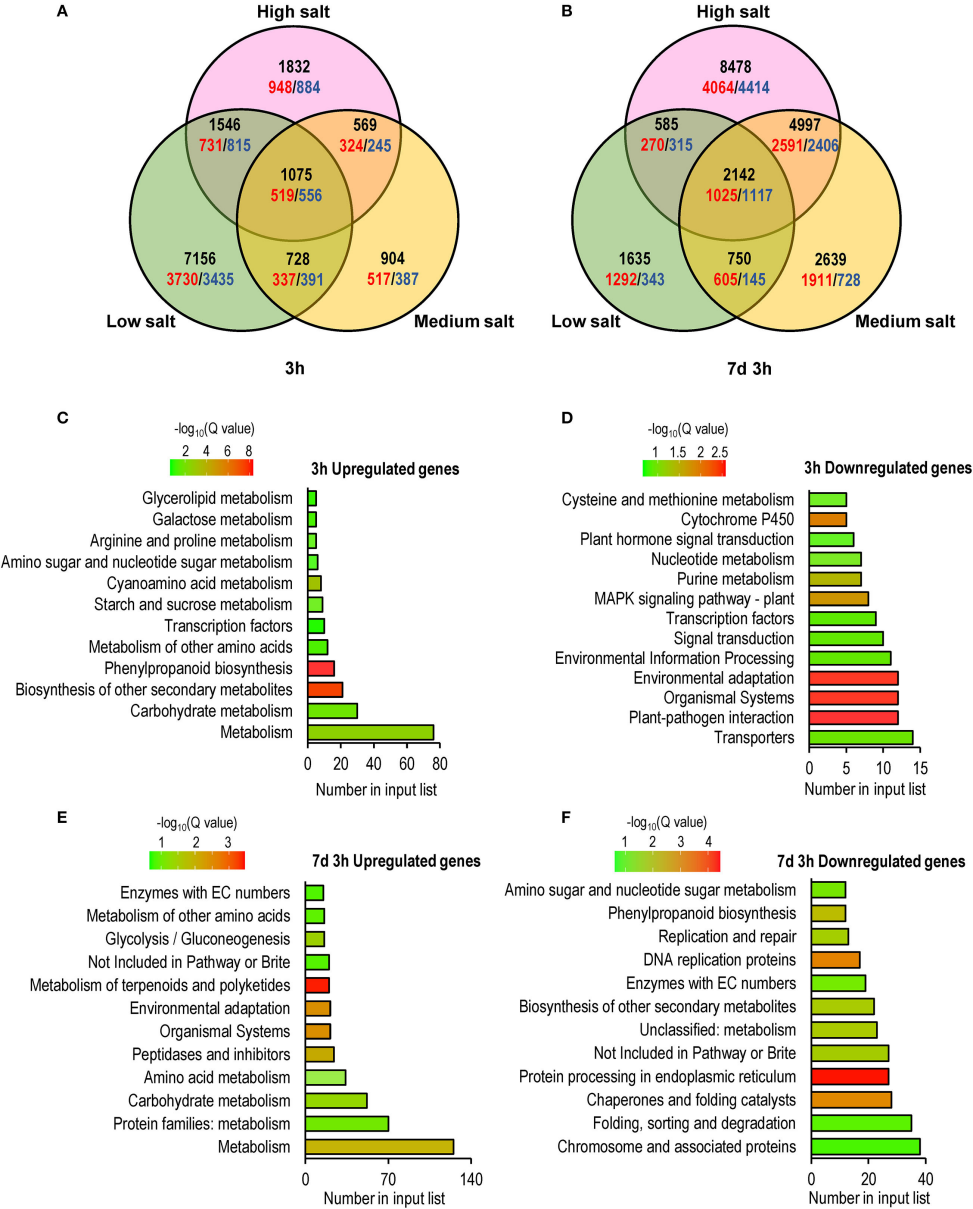
Global comparison of transcriptome profiling identified early and long-term salt response genes. **(A)** Venn diagrams indicating the numbers of early (3 h) and long-term (7 days and 3 h) salt-related genes compared to no salt control. The unregulated genes were colored red while downregulated genes were colored blue, and the total numbers were black. KEGG enrichment analysis of salt-response genes commonly regulated after 3h **(C,D)** or 7 days and 3h **(E,F)** treated in salt of all levels.

**Figure 6:**
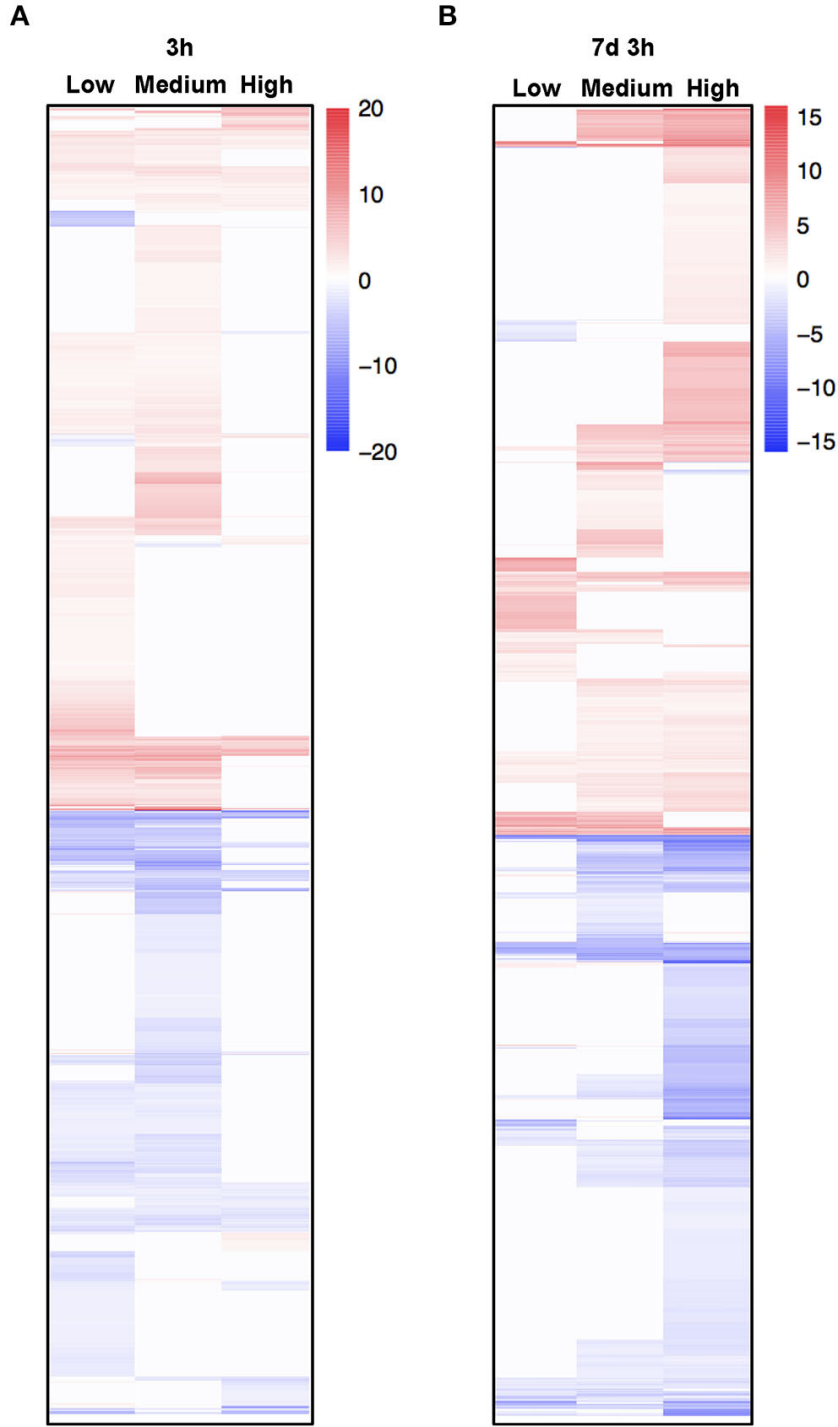
Heatmap of coregulated genes in response to different salt concentrations. **(A,B)** Heat map of differentially expressed genes (DEGs) commonly regulated under salt stress of all levels. The color scale in the heat map indicates the log-ratio of the normalized gene expression value in each sample to that in mock control.

To further explore the mechanism underlying the transcriptional response of *C. inerme* under short- and long-term salt stress, we performed RNA-sequencing (RNA-seq) to analyze the transcriptomes of *C. inerme* treated with the salt of different levels for 3 h (short-term) or 7 d plus 3 h (long-term). Although the physiological index was recorded on day 14 under the NaCl treatment, the transcriptome should be detected much earlier. It takes a certain time from transcriptional reprofiling, proteome remodeling, metabolic alterations, physiological changes, and cell differentiation for final morphological transformation. The commonly regulated salt-responsive genes were selected based on the following criteria: genes are up- or downregulated (|log2|≥ 1, adjusted *p* < 0.05) by salinity application compared to no salt control. Under short-term exposure, the transcripts of 10,505, 3,276, and 5,022 genes were altered by low, medium, and high salinity, respectively (Figure 5A, Supplementary Table S3). In contrast, under long-term stress, there were 5,112, 10,528, and 16,202 genes differentially regulated by low, medium, and high salinity, respectively (Figure 5A; Supplementary Table S4). Only 1,075 and 2,142 genes were coregulated by different levels of NaCl treatment for short-term and long-term, respectively (Figures 5B, 6A,B). The above data revealed that *C. inerme* may reprogram divergent transcriptional machineries in adaption to distinct salinity conditions.

For the short-term salt treatment, KEGG enrichment analysis revealed that salinity-induced genes were mainly involved in the metabolism of carbohydrates and secondary metabolites, such as phenylpropanoid, amino acids, starch and sucrose, cyanoamina acid, proline, and glycerolipid (Figure 5C). Phenylpropanoid is an important secondary metabolite and plays a crucial role in response to stimuli and stresses (Ignat et al., 2011). Starch and sugar provide energy to support plant growth under stress and function as osmoprotectants to mitigate the negative effect of the stress (Dong and Beckles, 2019). Elevated expression of phenylpropanoid, proline, starch, and sugar metabolism-related genes suggested that *C. inerme* produces abundant secondary metabolites and increases energy supply in defense of early salt injury. In contrast, salinity-repressed genes were mainly related to transporters, organismal systems, nucleotide metabolism, and plant hormone signal transduction (Figure 5D), indicating *C. inerme* rapidly slows down its growth at the early salt stress stage.

For the long-term salinity acclimation, the genes involved in the metabolism of amino acids, proteins, and carbohydrates, as well as organismal systems and environmental adaption were upregulated (Figure 5E), suggesting that *C. inerme* promotes comprehensive cellular processes in adaption to continuous salt stress. In contrast, the genes related to cellular processes, such as DNA replication, DNA repair, protein processing, folding, sorting, and degradation were downregulated (Figure 5F), revealed that *C. inerme* reduces its cellular energy consumption under continuous salt conditions.

**Figure 7:**
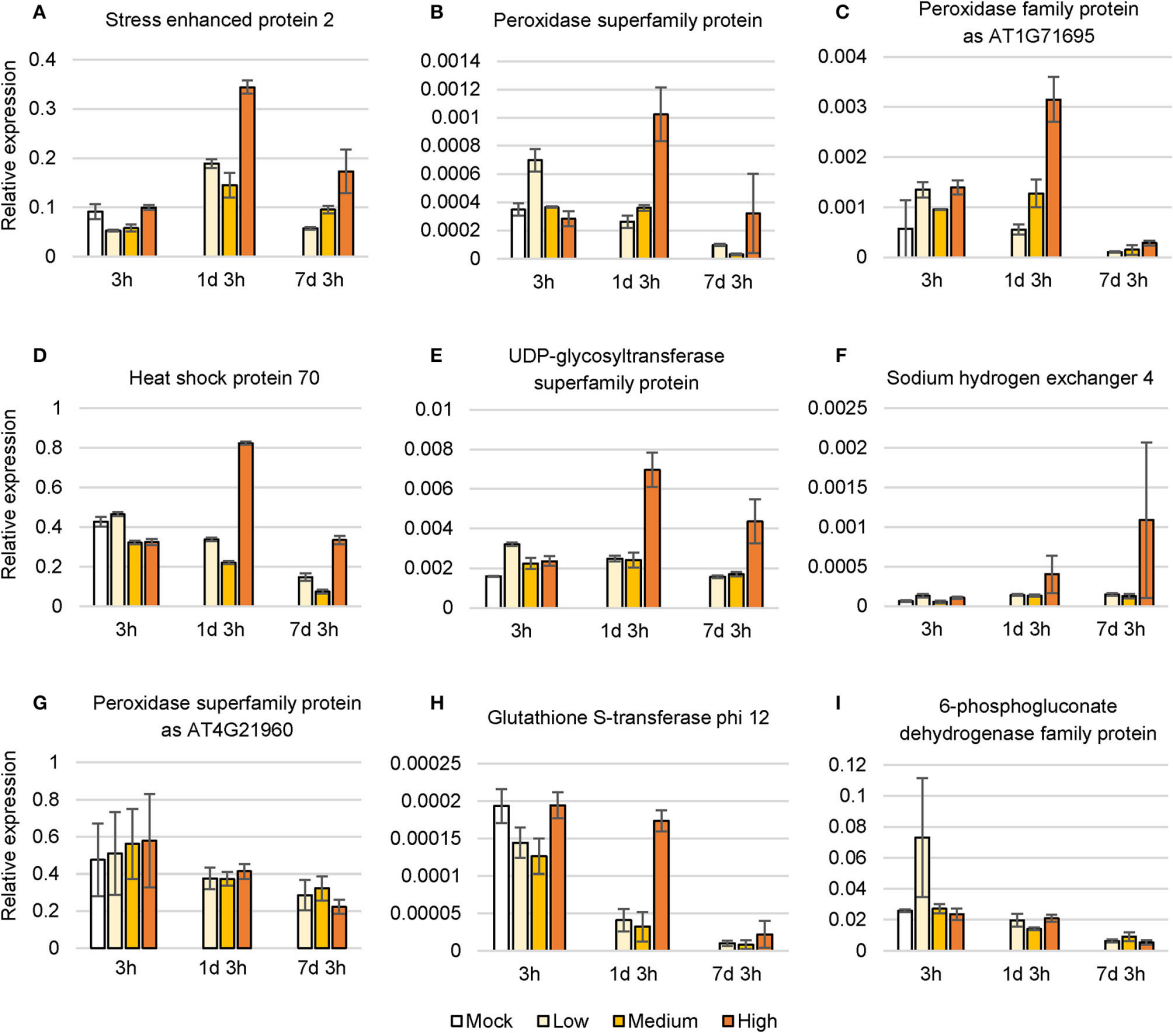
Relative expression level of nine salt response genes under NaCl stress. **(A–I)** Differentially expressed genes under different saline concentration for the indicated time. *H*_3_ was used as a normalization control. Data are the means of three technical replicates ± SD. Mock, no salt control; Low, low salt stress; Medium, medium salt stress; High, high salt stress.

To confirm the changes in expression of salt-induced genes, we conducted qRT-PCR with *C. inerme* young leaves under a simple time course of salinity treatment. As expected, genes involved in the environmental response, metabolism, and ion exchange were upregulated after NaCl application for 1 day, yet finally decreased after 7 days under high level of salt (Figures 7A–F). While, other metabolite-related genes were downregulated in general at all levels of NaCl for these indicated days (Figures 7G–I). In summary, the results suggested that *C. inerme* shifts from salt acclimation to salt tolerance by metabolic readjustment *via* the analysis of transcriptional profile.

## Discussion

The woody shrub *C. inerme* belongs to the Lamiaceae family, whose natural habitats are coast, beach, and tide with salt-tolerant ability. However, how *C. inerme* displays its developmental plasticity to adjust the salinized soils remains unclear. In this study, we provide evidence showing that *C. inerme* trim its growth at different levels to adapt to the saline environment although suffered from salt stress. First, phenotypic analyses revealed that premature senescence and biomass reduction are results from resources redirected to improve the tolerance to saline adversity. Secondly, that the increase of MDA and the decrease of GSH indicates the damage was gradually serious, yet the boosting of Pro under medium and high salt treatment demonstrated that *C. inerme* relocates energy for produced osmotic-resistant metabolite to survive. Third, ROS scavenging enzymes climbed gradually from no salt to medium salt treatment but declined significantly under high salt, which explains that the saline tolerance system is robust under the medium level of salt stress yet overloads when faced with high salt adversity. Fourth, enhanced chlorophyll and ionic fluxes content increases photosynthesis, whereas declined photosynthesis efficiency reflects the tolerance mechanism, in which *C. inerme* tries to increase photosynthesis to produce energy for resistance and make up for the damage caused by the elevated salt environment. Finally, global transcriptional profiling revealed that *C. inerme* switched on osmoprotection mainly to modulate the expression of genes related to metabolism to maintain cellular osmotic homeostasis and retard growth under salt stress. These findings thus uncover how *C. inerme* survive and strive in the salinized circumstance with salt-tolerant ability.

The plant actively retards the growth rate in response to salt stress, leading to increased survival. Thus, a positive relationship between reduced biomass and elevated NaCl concentration has been observed in this long-term salt stress experiment (1~2 weeks here), although the chlorophyll content increased yet photosynthetic efficiency declined (Figures 1C,D, 2). Salt stress activates ethylene biosynthesis and signaling, which reduced photosynthesis in young leaves while accelerating plant senescence (Ceusters and Van de Poel, 2018). Likewise, the etiolation and crease of leaves were more and more obvious with the increase of salt (Figure 1A). These results agree with the general observation that after being stressed, photosynthetic efficiency will rapidly decrease, whether it is salt-sensitive or salt-tolerant plants.

Dramatic accumulation of Pro is a conventional physiological response of plants in the face of various abiotic stresses. Pro effectively maintains cell turgor, promotes water flow into the cytoplasm, stabilizes the active conformation of enzyme proteins, preserves cell pH value, and eliminates free radicals and redox potential (Kaur and Asthir, 2015). There is a general consensus that the increase of proline content following stress is beneficial for the plant (Verbruggen and Hermans, 2008). In particular, Pro indirectly protects the photochemical efficiency of PSII as well as directly scavenges ROS during drought (Molinari et al., 2007). Here, we found that Pro stayed at a low level in the presence of no or low salt treatment, but rocketed under medium and high saline stress (Figure 2B). Accordingly, the actual photochemical quantum efficiency ΦPSII decreased gently with the increase of salt concentration (Figure 3H). These results agree with the previous discovery that Pro protects the PSII reaction center from damage, although PSII is somehow destroyed and its potential activity is inhibited under high salt stress.

It is generally believed that salt stress can increase the production of ROS such as singlet oxygen (1O^2^), superoxide radical (O^2−^), hydrogen peroxide (H_2_O_2_), and hydroxyl radical (OH^−^), which seriously disrupt normal metabolism in the plant through oxidation of membrane lipids, proteins, and nucleic acids (Smirnoff, 1993; Hernandez et al., 2001). On the other hand, plants evolve a resistance system including antioxidant enzymes such as SOD, CAT, and POD to protect their cells against ROS. Some studies suggest that salt stress leads to an increase in SOD activity in salt-tolerant plants but a decrease in salt-sensitive ones (Rout and Shaw, 2001). Meanwhile, POD and CAT can catalyze H_2_O_2_ into H_2_O and O_2_ to prevent the continuous accumulation of ROS and decelerate the senescence of the plant. In this study, no significant changes in SOD and POD were observed, probably due to the 2-week long-term stress condition when the intracellular homeostasis already reached before then (Figures 2E,F). Little change was also detected in GSH content, which participates in the elimination of H_2_O_2_ through the catalysis of NADPH-dependent glutathione reductase and can be used as an antioxidant (Figure 2C).

Various indicators of photosynthesis showed that the photosynthetic rate of *C. inerme* decreased significantly under medium and high salt treatment (Figures 3B–H), but the chlorophyll content was higher than that at no or low salt concentration (Figure 3A). It can be explained that *C. inerme* attempts to synthesize more chlorophyll through the metabolic pathway to improve photosynthetic efficiency and produce energy to make up for the energy loss to resist salt stress. However, mainly due to the damage to chloroplast structure or the destruction of enzymes required for photosynthesis, the overall photosynthetic efficiency in *C. inerme* was decreased under medium and high salinity, which reflects the destructive effect of the salt environment.

Various experimental results support the notion that Na^+^/H^+^ antiporters in the plasma membrane mediate Na^+^ effluxes (Qiu et al., 2002, 2003; Xiong et al., 2002), whose proton motive force is offered by P-type H^+^-ATPases (Schachtman and Liu, 1999). In this study, the outflow of Na^+^ and K^+^ increased significantly from mesophyll cells to balance the passive absorption of excess Na^+^ by the plant under high NaCl concentration (Figures 4A–D). However, the influx of H^+^ stagnated as more H^+^ participated in the function of H^+^-ATPase and flew out (Figures 4E,F). Finally, the activity of H^+^-ATPase was increased (Figure 4G), which is in agreement with other studies mentioned. The results suggested that *C. inerme* copes with the absorption of excessive Na^+^ caused by salt stress by increasing the outflow of cations by the motive force from H^+^-ATPase.

High throughput and transcriptome analysis are important for salt-stress-related gene screening and effective approaches for studying the molecular regulation of salt tolerance in the plant (Song et al., 2020). Results of this study showed that *C. inerme* adapted to salinity stress by adjusting the metabolism of nucleotides, amino acids, carbohydrates, and enzymes along with transcriptional factors and plant hormones at the early stage (Figures 5–7). These findings are in agreement with and include the discovery shown in the previous RNA-Seq study on *C. inerme*, which demonstrated that genes related to plant hormone signaling were crucial to the saline response of *C. inerme* (Xiong et al., 2019). However, decreased expression of genes in the cellular process and increased expression of genes related to metabolism were detected, indicating the survival strategy of *C. inerme* by constitutively modulating metabolism to meet the challenges of ion disorders and the toxic effect caused by NaCl adversity.

## Materials and Methods

### Plant Materials and Growth Conditions

Seedlings of *C. inerme* were planted in the local red soil in a greenhouse in South China Botanical Garden, Chinese Academy of Sciences in Guangzhou City, Guangdong Province. The 30-day-old *C. inerme* seedlings were transferred to the coral sand which was collected from Paracel Islands. Before transplanting, the coral sands were pre-washed five times to a final pH at 9.35, electrical conductivity at 0.170 ms/cm, and mass fraction of salt at 0.319. After transplanting, seedlings were irrigated with deionized water supplemented with 1 g/L water-soluble fertilizer Huawuque containing 20% N, 20% P, and 20% K, every 3 days. Then, 7 days after acclimation, the seedlings were treated with 0.1, 0.3, and 0.5 M NaCl solution, respectively. To avoid the adverse effects of rapid change in soil salt content, NaCl was gradually added to the final concentration on the 7th day (Supplementary Table S1). The control group was irrigated with deionized water. Five replicates were set for each treatment. After treatment, the 3^rd^ and 4^th^ newly grown leaves were harvested for physiology and gene expression analysis. All enzyme extract procedures were conducted at 4°C.

### Phenotyping and Biomass Analysis

Forteen days after salt treatment, the plant height, fresh weight, and dry weight of the seedlings were measured. The fresh weight was determined after the root, stem, and leaf of the seedlings were fully washed. For dry weight analysis, clean seedlings were placed at 105°C for 30 min, then dried to constant weight at 80°C.

### MDA Analysis

The 0.5 g leaves were ground and homogenized in 10% cold TCA buffer. After centrifuging for 10 min, the supernatant was collected and 0.5% TBA was added. After being boiled for 20 min, the supernatant was collected and the OD values were tested at 450, 532, and 600 nm, respectively.


$$\text{MDA}(\mu \text{mol/g})\;=\frac{{\left[6.45(OD_{532}-OD_{600})-0.56OD_{450}\right]}^{*}V}{W}$$

OD_450_, OD_532_, and OD_600_, absorbance values at 450, 532, and 600 nm, respectively; V, volume of the supernatant (ml); W, fresh weight of the plant tissue (g).

### Proline Analysis

The 0.3 g leaves were ground and homogenized in 2 mL 80% ethanol, and then placed in 80^o^C water bath for 20 min. After filtering, 2 mL glacial acetic acid and 2 mL ninhydrin were added to 2 mL homogenate. After boiling for 15 min, the mixture was cooled to room temperature and the OD value at 520 nm was measured. A standard curve was established with pure proline as reference.


$$\text{Pro}(\text{mg/g})=\frac{C{\;}^{*}\;V}{1000M}$$

V, volume of the homogenate (mL); C, proline content analysis using standard curve as reference (g/mL); M, fresh weight of the leaf sample (g).

### GSH Analysis

The 0.5 g tissue was ground and homogenized in 5 mL 5% trichloroacetic acid. After centrifuging at 15,000 rpm for 10 min, 0.25 mL supernatant was added with 2.6 mL 150 mM PBS (pH7.7) and 0.15 mL DTNB (75.3 mg DTNB dissolved in 30 mL 100 mM PBS pH6.8). The OD value was determined at 412 nm and the content of GSH was calculated according to the standard curve made with pure GSH.

### Total Flavonoids Analysis

The 0.5 g leaves were ground and homogenized in 5 mL 70% ethanol. The homogenate was shattered by ultrasonic for 30 min and rotated for 12 h at room temperature. After centrifuging at 12,000 rpm for 10 min, 300 μL supernatant was added with 7 mL 70% ethanol, 500 μL 5% NaNO_2_ solution, and stayed at room temperature for 6 min. The 500 μL of 10% Al (NO_3_)_3_ was then added and placed at room temperature for 6 min. Finally, 4 mL 4% NaOH and 2 mL 70% ethanol were added and stayed still for 15 min. The OD value at 510 nm was determined and the content of total flavonoids was calculated according to the standard curve. The standard curve was established by diluting pure rutin with a 70% ethanol solution.

### SOD Activity Analysis

About 0.5 g of leaves were ground and homogenized with 5 mL cold PBS buffer. The homogenate was centrifuged at 10,000 rpm for 15 min. 50 μL supernatant was mixed with the reaction solution including 0.05 M PBS, 220 mM Met, 1.25 mM NBT, and 33.5 mM riboflavin, and placed under light for 20 min. The mixture of PBS buffer and reaction solution without plant homogenate which was kept in the dark was used as a control group. The OD value of the reaction was measured at 560 nm.


$$\text{SODactivity}(\text{U/g})=\frac{(A_{\text{CK}}-A_{\text{E}})*V}{A_{\text{CK}}*0.5*W*V}$$

A_CK_, OD value of the control group; A_E_, OD value of the sample; V, volume of enzymic reaction mixture; V_t_, sample volume added to reaction solution; W, fresh weight of plant leaves (g).

### POD Activity Analysis

About 0.2 g of leaves were added with five times the volume of PBS pH 7.0 by mass concentration in a precooled 5-ml tube and ground in the 60 Hz grinder for 2 min. The homogenate was centrifuged at 15,000 rpm for 15 min at 4^o^C. And 50 μl supernatant was mixed with 1 mL 0.3% H_2_O_2_, 0.95 mL guaiacol, and 1 mL PBS pH7. The OD value at 470 nm was then measured every 10 s for 1 min. The 0.01 increase of OD value per minute was defined as one enzyme activity unit.

### Chlorophyll Content Analysis

About 0.4 g of leaves were cut into pieces and extracted with the extraction buffer containing acetone, anhydrous ethanol, and deionized water in a ratio of 4.5:4.5:1. The extract stayed at 4°C for 24 h. OD value of the mixture was taken at 645 nm and 663 nm, and the chlorophyll content of plant leaves was then calculated according to the formula below.


$$\begin{array}{c} \text{Chla}(\text{mg/g})=\frac{12.7OD_{663}-2.69OD_{645}*V}{1000W}\\ \text{ Chlb}(\text{mg/g})\;=\frac{-22.9OD_{645}-4.68OD_{663}*V}{1000W} \end{array}$$

Total chlorophyll (mg/g) = Chla + Chlb

V, final volume of the extract (mL); W, fresh weight of leaves (g).

### Determination of Photosynthetic Index

The net photosynthetic rate, transpiration rate, stomatal conductance, and intercellular CO_2_ concentration from the 3^rd^ and 4^th^ leaves of plants were measured *via* portable photosynthetic instrument Li-6800 (LI-COR, USA) under cloudless sunny weather with unfolded greenhouse canopy as well as avoiding the morning and evening time with strong and weak sunlight.

### Determination of Chlorophyll Fluorescence

The plants were placed in dark for more than 3 h. The maximum photochemical efficiency (Fv/Fm), initial fluorescence (F0), and photochemical quantum efficiency of (ΦPSII) from the 3^rd^ and 4^th^ leaves of plants were then measured by portable photosynthetic instrument Li-6800 (LI-COR, USA).

### Concentration, Direction, and Velocity of K^+^, H^+^, and Na^+^ Flow in Mesophyll Cells

A 0.5-mm diameter disc dissected from the 3^rd^ and 4^th^ newly grown leaves after 14 days of salinity treatment were harvested and placed on buffer (100 mM KCl for K^+^; 15 mM NaCl, 40 mM KH_2_PO_4_ pH 7.0 for H^+^; 250 mM NaCl for Na^+^) in the petri dish. A pair of needle tip-like microsensors controlled by the software were inserted into both sides of the mesophyll cell membrane under a microscope. Voltage signals were captured to reflect the ionic fluxes between the cytoplasm and the intercellular space. The concentration, direction, and velocity of K^+^, H^+^, and Na^+^ ion flow were then detected *via* selective microsensors. This micro measurement technology was provided by Xuyue (Beijing) Scientific Technology Company and the technical service center of Jiangsu Normal University (BIO-IM Series, Younger USA LLC, Amherst, Ma 01002).

### H^+^-ATP Synthase Activity

The activity of H^+^-ATP synthase was determined by the ELISA kit (CUSABIO BIOTECH Co., Ltd., Wuhan, China) according to the manufacturer's instructions. In brief, 0.2 g leaves were added with nine times the volume of PBS pH 7.4 by mass concentration in a precooled 5-ml tube and ground in the 60 Hz grinder for 2 min. The homogenate was centrifuged at 15,000 rpm for 15 min at 4^o^C. About 10 μL reference standard or 40 μL supernatant sample was added into the well plate with the micropores pre-coated with plant hydrogen ATPase (H^+^-ATP) antibody. HRP labeled antibody was successively added and washed thoroughly. The substrate TMB used for color development was added to convert into blue under the catalysis of peroxidase and finally into yellow by the presence of acid. The color depth was positively correlated with the synthase activity in the sample. OD value of the sample was taken at 450 nm and the activity was calculated.

### RNA-Seq Analysis

The total RNA was extracted using Column Plant RNAout2.0 (Tiandz Inc., Beijing, China) according to the manufacturer's protocol. Extracted RNA was treated with DNase (Tiandz Inc., Beijing, China) to remove genomic DNA. The sequencing libraries with an average insert length of 240 bp were generated using the NEBNextR UltraTM Directional RNA Library Prep Kit for IlluminaR (NEB, USA) and sequenced on the Illumina HiSeq4000 platform (Illumina Inc., USA) by Bio&Data Biotechnology Co., Ltd (Guangzhou, China) following manufacturer's recommendations. The reads were assembled into unigenes which were aligned with the *Arabidopsis thaliana* genome by BLASTN for functional annotation since the genomic information of *Clerodendrum inerme* was not yet available. Differentially expressed genes were defined based on the following criteria: a DESeq2 adjusted *p* < 0.05 and fold change > 2 compared with the control samples. The hypergeometric Fisher exact test (*p* < 0.01) and Benjamini (FDR < 0.05) were performed to detect statistically significant enrichment of the KEGG pathway. All sequence data were uploaded into the BioProject database hosted by the National Center for Biotechnology Information (NCBI) under the BioProject PRJNA817709. Three independent replicates were performed in this experiment.

### RNA Extraction and qRT-PCR

Total RNA was extracted by a HiPure Plant RNA Mini Kit (Magen, China). The cDNA was synthesized by HiScript® III All-in-one RT SuperMix Perfect for qPCR (Vazyme, China). The qRT-PCR reaction was performed in a 384-well block using a ChamQ Universal SYBR qPCR Master Mix (Vazyme, China). The values for each set of primers were normalized relative to the *H3* gene. All qRT-PCR reactions were performed in triplicates. The specific primers used in this study are listed in Supplementary Table S2.

## Conclusion and Future Perspectives

The response of *C. inerme* to salt stress begins to show under a low level of salt stress. Membrane lipid peroxidation is manifested in the early stage, and it responds to saline stress through antioxidant enzymes, osmotic regulators, and antioxidants, which is consistent with the finding of transcriptional global profiling. The photosynthetic rate of *C. inerme* was also affected; PSII can maintain a certain efficiency under low and medium salt stress, but it is aggrieved under high salt stress. In the face of NaCl stress, the outflow of Na^+^ tends to decrease and the influx of H^+^ decreases at low and medium saline levels, which may be due to the induction of the Na^+^/H^+^ antiporter of the tonoplast membrane by H^+^-ATPase to store Na^+^ store in vacuoles. Thus the accumulation of Na^+^ reduces in the cytoplasm. All these biochemical and physiological results show that *C. inerme* suffers ion toxicity under high salt stress. Moreover, efforts should be made in the future to investigate the roles of salt tolerance of *C. inerme* through a multi-omics approach. Together, the findings can provide a rich resource for breeding salt-tolerant cultivars through biotechnological measures of the time.

## Data Availability Statement

The datasets presented in this study can be found in online repositories. The names of the repository/repositories and accession number(s) can be found below: National Center for Biotechnology Information (NCBI) BioProject database under accession number PRJNA817709.

## Author Contributions

XL and FW designed the research. ML, FH, DX, ZC, QZ, QX, FZ, and DL performed the experiments. ML, DX, SJ, and HC analyzed the data. ML and XL wrote the manuscript. All authors contributed to the article and approved the submitted version.

## Funding

This work was supported by the National Natural Science Foundation of China (32070551), the Science and Technology Planning Project of Guangdong Province (2021B1212110004), the Strategic Priority Research Program of the Chinese Academy of Sciences (XDA13020603), and the Urban Forestry Plan of Guangzhou (2021-120).

## Conflict of Interest

The authors declare that the research was conducted in the absence of any commercial or financial relationships that could be construed as a potential conflict of interest.

## Publisher's Note

All claims expressed in this article are solely those of the authors and do not necessarily represent those of their affiliated organizations, or those of the publisher, the editors and the reviewers. Any product that may be evaluated in this article, or claim that may be made by its manufacturer, is not guaranteed or endorsed by the publisher.

## Supplementary Material

Supplementary Figure 1 Morphogenesis of *C. inerme* before salinity stress. **(A)** Shoot height was measured on day 0 under NaCl stress. **(B)** Root-to-shoot ratio by dry weight after 14 days under different saline condition. Different letters indicate significant differences among plant groups (*p* < 0.05, Mann Whitney test). Mock, no salt control; L, low salt stress; M, medium salt stress; H, high salt stress.

Supplementary Table S1 NaCl concentration for salinity stress.

Supplementary Table S2 Primers used for qRT-PCR.

Supplementary Table S3 Alternatively expressed transcripts under short-term salt exposure.

Supplementary Table S4 Alternatively expressed transcripts under long-term salt exposure.

## References

[B1] Acharya B. R. Sandhu D. Duenas C. Ferreira J. F. S. Grover K. K. (2022). Deciphering molecular mechanisms involved in salinity tolerance in Guar (*Cyamopsis tetragonoloba* (L.) Taub.) using transcriptome analyses. Plants (Basel) 11, 291. 10.3390/plants11030291 35161272 PMC8838131

[B2] Bose J. Munns R. Shabala S. Gilliham M. Pogson B. Tyerman S. D. (2017). Chloroplast function and ion regulation in plants growing on saline soils: lessons from halophytes. J. Exp. Bot. 68, 3129–3143. 10.1093/jxb/erx142 28472512

[B3] Ceusters J. Van de Poel B. (2018). Ethylene exerts species-specific and age-dependent control of photosynthesis. Plant Physiol. 176, 2601–2612. 10.1104/pp.17.01706 29438047 PMC5884594

[B4] Chen C. X. Shang X. L. Sun M. Y. Tang S. Y. Khan A. Zhang D.. (2022). Comparative transcriptome analysis of two sweet sorghum genotypes with different salt tolerance abilities to reveal the mechanism of salt tolerance. Int. J. Mol. Sci. 23, 2272. 10.3390/ijms23042272 35216389 PMC8877675

[B5] Dietz K. J. Jacob S. Oelze M. L. Laxa M. Tognetti V. de Miranda S. M.. (2006). The function of peroxiredoxins in plant organelle redox metabolism. J. Exp. Bot. 57, 1697–1709. 10.1093/jxb/erj160 16606633

[B6] Dong S. Y. Beckles D. M. (2019). Dynamic changes in the starch-sugar interconversion within plant source and sink tissues promote a better abiotic stress response. Plant Physiol. 234, 80–93. 10.1016/j.jplph.2019.01.007 30685652

[B7] Flowers T. J. Munns R. Colmer T. D. (2015). Sodium chloride toxicity and the cellular basis of salt tolerance in halophytes. Ann. Bot. 115, 419–431. 10.1093/aob/mcu217 25466549 PMC4332607

[B8] Glenn E. P. Brown J. J. Blumwald E. (1999). Salt tolerance and crop potential of halophytes. Crit. Rev. Plant Sci. 18, 227–255. 10.1080/07352689991309207

[B9] Hernandez J. A. Ferrer M. A. Jimenez A. Barcelo A. R. Sevilla F. (2001). Antioxidant systems and O^−2(.−)^/H_2_O_2_ production in the apoplast of pea leaves. Its relation with salt-induced necrotic lesions in minor veins. Plant Physiol. 127, 817–831. 10.1104/pp.010188 11706165 PMC129254

[B10] Ignat I. Volf I. Popa V. I. (2011). A critical review of methods for characterisation of polyphenolic compounds in fruits and vegetables. Food Chem. 126, 1821–1835. 10.1016/j.foodchem.2010.12.026 25213963

[B11] James R. A. Davenport R. J. Munns R. (2006). Physiological characterization of two genes for Na^+^ exclusion in durum wheat, *Nax1* and *Nax2*. Plant Physiol. 142, 1537–1547. 10.1104/pp.106.086538 17028150 PMC1676036

[B12] Kaur G. Asthir B. (2015). Proline: a key player in plant abiotic stress tolerance. Biol. Plantarum 59, 609–619. 10.1007/s10535-015-0549-3

[B13] Khan M. A. Qaiser M. (2006). Halophytes of Pakistan: characteristics, distribution and potential economic usages, in Sabkha Ecosystems. Tasks for Vegetation Science, Vol. 42, eds Khan M. A. Böer B. Kust G. S. Barth H. J. (Dordrecht: Springer). 10.1007/978-1-4020-5072-5_11

[B14] Li L. Li M. M. Qi X. W. Tang X. L. Zhou Y. F. (2018). *De novo* transcriptome sequencing and analysis of genes related to salt stress response in Glehnia littoralis. PeerJ 6, e5681. 10.7717/peerj.5681 30294511 PMC6170154

[B15] Lotfi N. Vahdati K. Amiri R. Kholdebarin B. (2010). Drought-induced accumulation of sugars and proline in radicle and plumule of tolerant walnut varieties during germination phase. Acta. Hortic. 861, 289–295. 10.17660/ActaHortic.2010.861.39

[B16] Meloni D. A. Oliva M. A. Martinez C. A. Cambraia J. (2003). Photosynthesis and activity of superoxide dismutase, peroxidase and glutathione reductase in cotton under salt stress. Environ. Exp. Bot. 49, 69–76. 10.1016/S0098-8472(02)00058-832957907

[B17] Mittler R. Vanderauwera S. Gollery M. Van Breusegem F. (2004). Reactive oxygen gene network of plants. Trends. Plant Sci. 9, 490–498. 10.1016/j.tplants.2004.08.009 15465684

[B18] Molinari H. B. C. Marur C. J. Daros E. de Campos M. K. F. de Carvalho J. F. R. P. Bespalhok J. C.. (2007). Evaluation of the stress-inducible production of proline in transgenic sugarcane (*Saccharum* spp.): osmotic adjustment, chlorophyll fluorescence and oxidative stress. Physiol. Plantarum 130, 218–229. 10.1111/j.1399-3054.2007.00909.x

[B19] Munns R. Gilliham M. (2015). Salinity tolerance of crops - what is the cost? New Phytol. 208, 668–673. 10.1111/nph.13519 26108441

[B20] Park H. J. Kim W. Y. Yun D. J. (2016). A new insight of salt stress signaling in plant. Mol. Cells 39, 447–459. 10.14348/molcells.2016.0083 27239814 PMC4916396

[B21] Qiu Q. S. Barkla B. J. Vera-Estrella R. Zhu J. K. Schumaker K. S. (2003). Na+/H+ exchange activity in the plasma membrane of Arabidopsis. Plant Physiol. 132, 1041–1052. 10.1104/pp.102.010421 12805632 PMC167042

[B22] Qiu Q. S. Guo Y. Dietrich M. A. Schumaker K. S. Zhu J. K. (2002). Regulation of SOS1, a plasma membrane Na+/H+ exchanger in Arabidopsis thaliana, by SOS2 and SOS3. Proc. Natl. Acad. Sci. U. S. A 99, 8436–8441. 10.1073/pnas.122224699 12034882 PMC123085

[B23] Rad P. B. Roozban M. R. Karimi S. Ghahremani R. Vahdati K. (2021). Osmolyte Accumulation and Sodium Compartmentation Has a Key Role in Salinity Tolerance of Pistachios Rootstocks. Agriculture 11, 708. 10.3390/agriculture11080708

[B24] Rout N. P. Shaw B. P. (2001). Salt tolerance in aquatic macrophytes: possible involvement of the antioxidative enzymes. Plant Sci. 160, 415–423. 10.1016/S0168-9452(00)00406-4 11166427

[B25] Schachtman D. Liu W. (1999). Molecular pieces to the puzzle of the interaction between potassium and sodium uptake in plants. Trends. Plant Sci. 4, 281–287. 10.1016/S1360-1385(99)01428-4 10407444

[B26] Schroeder J. I. Delhaize E. Frommer W. B. Guerinot M. L. Harrison M. J. Herrera-Estrella L.. (2013). Using membrane transporters to improve crops for sustainable food production. Nature 497, 60–66. 10.1038/nature11909 23636397 PMC3954111

[B27] Skorupa M. Golebiewski M. Domagalski K. Kurnik K. Abu Nahia K. Zloch M.. (2016). Transcriptomic profiling of the salt stress response in excised leaves of the halophyte Beta vulgaris ssp maritima. Plant Sci. 243, 56–70. 10.1016/j.plantsci.2015.11.007 26795151

[B28] Smirnoff N. (1993). Tansley review. 52. The role of active oxygen in the response of plants to water-deficit and desiccation. New Phytol. 125, 27–58. 10.1111/j.1469-8137.1993.tb03863.x 33874604

[B29] Song Q. S. Joshi M. Joshi V. (2020). Transcriptomic analysis of short-term salt stress response in watermelon seedlings. Int. J. Mol. Sci. 21, 6036. 10.3390/ijms21176036 32839408 PMC7504276

[B30] Takahashi M. Asada K. (1988). Superoxide production in aprotic interior of chloroplast thylakoids. Arch. Biochem. Biophys. 267, 714–722. 10.1016/0003-9861(88)90080-X 2850770

[B31] Verbruggen N. Hermans C. (2008). Proline accumulation in plants: a review. Amino Acids 35, 753–759. 10.1007/s00726-008-0061-6 18379856

[B32] Xiong L. M. Schumaker K. S. Zhu J. K. (2002). Cell signaling during cold, drought, and salt stress. Plant Cell 14, S165–S183. 10.1105/tpc.000596 12045276 PMC151254

[B33] Xiong Y. P. Yan H. F. Liang H. Z. Zhang Y. Y. Guo B. Y. Niu M. Y.. (2019). RNA-Seq analysis of *Clerodendrum inerme* (L.) roots in response to salt stress. BMC Genomics 20, 724. 10.1186/s12864-019-6098-y 31601194 PMC6785863

[B34] Zhu J. K. (2002). Salt and drought stress signal transduction in plants. Annu. Rev. Plant. Biol. 53, 247–273. 10.1146/annurev.arplant.53.091401.143329 12221975 PMC3128348

[B35] Zhu J. K. (2003). Regulation of ion homeostasis under salt stress. Curr. Opin. Plant Biol. 6, 441–445. 10.1016/S1369-5266(03)00085-2 12972044

[B36] Zhu J. K. (2016). Abiotic stress signaling and responses in plants. Cell 167, 313–324. 10.1016/j.cell.2016.08.029 27716505 PMC5104190

