# Supplementary figures and images for: Physiological Measurements and Transcriptome Survey Reveal How Semi-mangrove Clerodendrum inerme Tolerates Saline Adversity
Source: Front Plant Sci. 2022 Jul 15;13:882884. doi: 10.3389/fpls.2022.882884 (PMC9337567; doi:10.3389/fpls.2022.882884)

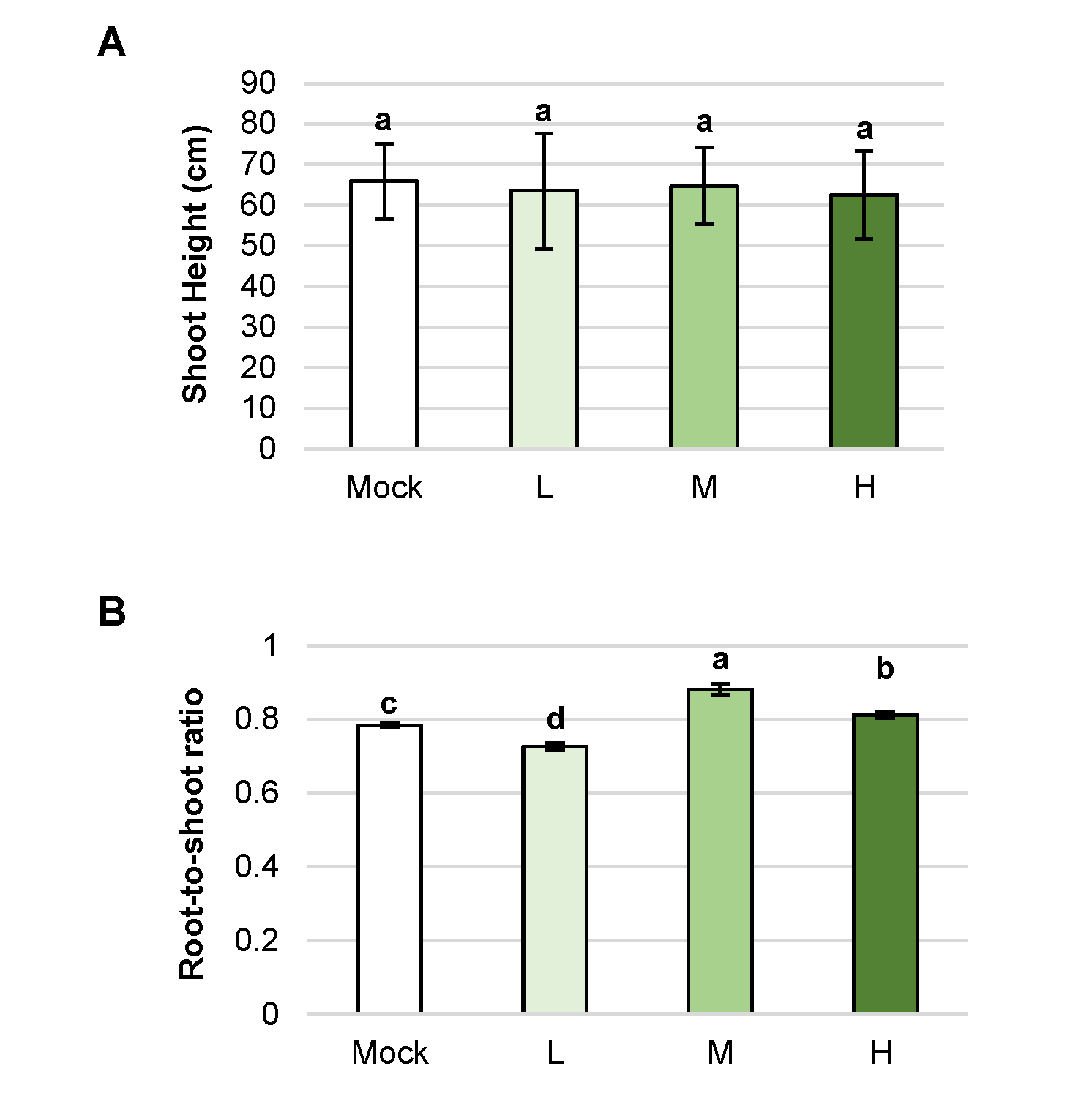

Supplement: Supplementary Figure 1 — Morphogenesis of C. inerme before salinity stress. (A) Shoot height was measured on day 0 under NaCl stress. (B) Root-to-shoot ratio by dry weight after 14 days under different saline condition. Different letters indicate significant differences among plant groups (p < 0.05, Mann Whitney test). Mock, no salt control; L, low salt stress; M, medium salt stress; H, high salt stress. [file Image_1.tiff]
